# Multiscale modeling of molecular structure and optical properties of complex supramolecular aggregates†

**DOI:** 10.1039/d0sc03110k

**Published:** 2020-10-01

**Authors:** Anna S. Bondarenko, Ilias Patmanidis, Riccardo Alessandri, Paulo C. T. Souza, Thomas L. C. Jansen, Alex H. de Vries, Siewert J. Marrink, Jasper Knoester

**Affiliations:** University of Groningen, Zernike Institute for Advanced Materials Groningen The Netherlands s.j.marrink@rug.nl j.knoester@rug.nl; University of Groningen, Groningen Biomolecular Sciences and Biotechnology Institute Groningen The Netherlands

## Abstract

Supramolecular aggregates of synthetic dye molecules offer great perspectives to prepare biomimetic functional materials for light-harvesting and energy transport. The design is complicated by the fact that structure–property relationships are hard to establish, because the molecular packing results from a delicate balance of interactions and the excitonic properties that dictate the optics and excited state dynamics, in turn sensitively depend on this packing. Here we show how an iterative multiscale approach combining molecular dynamics and quantum mechanical exciton modeling can be used to obtain accurate insight into the packing of thousands of cyanine dye molecules in a complex double-walled tubular aggregate in close interaction with its solvent environment. Our approach allows us to answer open questions not only on the structure of these prototypical aggregates, but also about their molecular-scale structural and energetic heterogeneity, as well as on the microscopic origin of their photophysical properties. This opens the route to accurate predictions of energy transport and other functional properties.

## Abstract 2

Multiscale modeling resolves the molecular structure of a synthetic light-harvesting complex, unraveling the microscopic origin of its photophysical properties.

## Graphical abstract

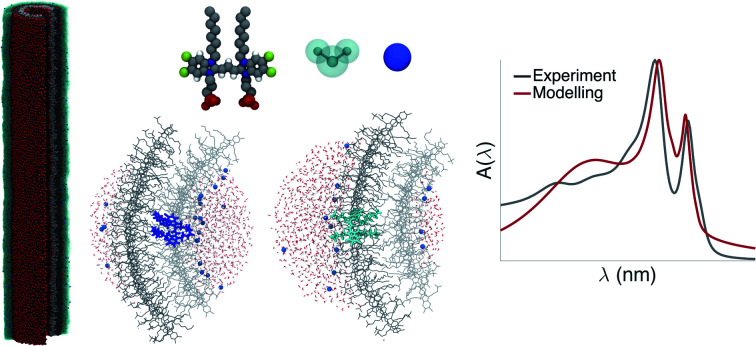

Supramolecular structures may self-assemble from a variety of building blocks, resulting in a wide range of advanced materials with attractive biomimetic, sensing, catalytic, optoelectronic and photonic functionalities.^1–10^ The close-packed nanoscale organization of the individual molecules within a supramolecular system, held together *via* noncovalent interactions, gives rise to the aggregate's (collective) properties. Assemblies consisting of dye molecules often exhibit unique collective optical properties and are of interest for opto-electronic applications as well as artificial light-harvesting complexes that mimic natural antenna systems of photosynthetic bacteria and plants.^11–13^ For example, chlorosomal antenna complexes of photosynthetic green sulfur bacteria are self-assembled into multilayer tubular structures having bacteriochlorophyll pigments as building blocks.^14–16^ The structure of these antenna complexes and the underlying molecular arrangement ensures that the process of light-harvesting and excitation energy transport is very efficient, even under extremely low light conditions.^17,18^ The quest to recreate such efficiency under laboratory conditions has sparked numerous studies of synthetic self-assembled systems mimicking natural chlorosomes, *e.g.* using porphyrins,^19^ zinc chlorin,^20^ and cyanine dyes.^21^ Of particular interest are the tubular aggregates of 3,3′-bis(2-sulfopropyl)-5,5′,6,6′-tetrachloro-1,1′-dioctylbenzimidacarbocyanine (C8S3).^22–25^ Cryo-TEM reveals a hierarchy of supramolecular architectures, including double-walled nanotubes; under certain conditions, bundles of nanotubes arise.^26^ Thus, this system allows for the occurrence of electronic excitation energy transport at various levels: within one wall, between walls of one tube, and between different tubes, similar to the situation in natural systems.^27,28^

To understand how such supramolecular systems work, as well as propose design rules for new materials, it is essential to determine the relationship between molecular structure and optical properties. Current experimental techniques, however, are unable to resolve the structure at the molecular level. This, in combination with the sensitivity of spectral properties to the details of the molecular packing, leads to a crucial role for theoretical modeling.^29^ For example, molecular dynamics (MD) simulations have been used to predict the molecular packing within a variety of supramolecular assemblies.^30–34^ However, synthetic amphiphiles with aromatic groups, such as cyanine dyes—often used to prepare aggregates with optical functionality—tend to fall into kinetic traps during spontaneous self-assembly simulations and the packing of the aromatic chromophores remains highly disordered on the accessible time scale, leading to predicted (optical) spectra that are not consistent with experimental data.^35^ This problem can be overcome by building assemblies based upon proposed architectures and assessing their stability in relatively short MD simulations.^36–38^ The drawback of this approach is the requirement of a thorough understanding of what to use as a starting point and how to validate the structure. In any case, proper validation requires the modeling of the optical spectra of the obtained structure, and finally, comparing it to the experiment. The demanding character of such methods explains why an important role is played by phenomenological modeling, in which a molecular packing is guessed and the optics is obtained from parametrizing an exciton model that describes the collective excited states of the assembly with interactions dictated by the guessed packing. By comparing the calculated spectra to experimental ones, the structure and exciton model may be fine-tuned. While this method has been successful in describing spectra,^23,39^ it is limited in its predictive power and also lacks access to essential microscopic parameters, such as tuning of the optical excitation energies imposed by the environment, disorder in these energies and structural heterogeneity.

In this work, we use an advanced multiscale approach to determine structure–optical property relationships for the C8S3 double-walled nanotubes, guided by comparison to experiments. The optical spectrum of these aggregates, in which multiple exciton peaks may be discerned, suggests a rather complex underlying molecular packing. This fact, combined with their sheer size going up to many thousands of molecules, makes these systems exceptionally challenging to resolve and leaves important questions concerning structure–function relationships unanswered or under debate, for instance the origin of the splitting between the two lowest-energy spectral bands.^23,38^ Here, we answer these questions by iteratively combining MD simulations to capture the details of molecular packing and structural disorder, an exciton Hamiltonian approach to calculate optical signatures, and explicit microelectrostatic calculations to estimate energetic disorder and solvent shifts. Previous attempts to reveal the structure of cyanine-based nanotubes were limited to small-scale system sizes,^37,38^ modeling optical features phenomenologically rather than using atomistic information^38^ or featuring simpler, single-walled systems.^37^ In addition to answering important questions for the C8S3 double-walled nanotubes, our study opens the way to explain and predict at an unprecedented level of detail the functional properties of other highly complex molecular materials.

## Results and discussions

### Conceptual workflow

**Scheme 1:**
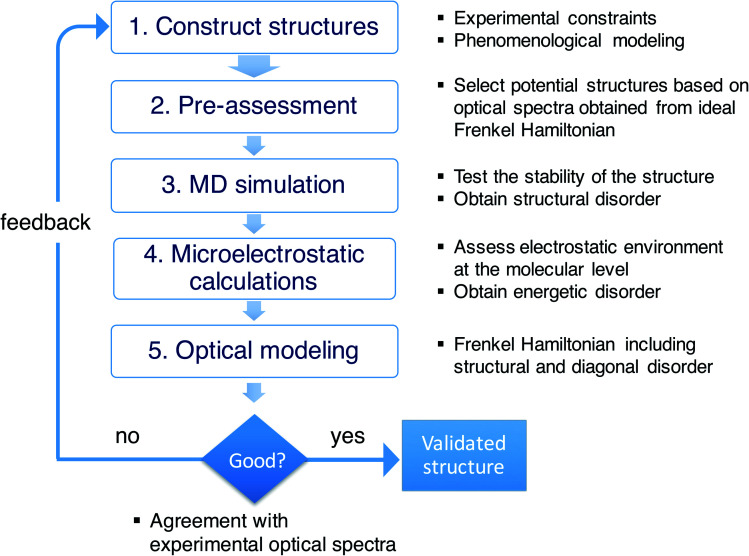
Overview of the workflow of the multiscale approach.

As stressed in the introduction, the type of aggregates of interest are too complex to allow for a direct (*ab initio*) modeling of their structure. Scheme 1 illustrates the overall multiscale approach that we follow to reveal the molecular packing of such complex supramolecular aggregates. In step 1, we construct perfectly ordered starting structures exploring the structural parameters within the available experimental constraints. In step 2, we pre-assess the optical properties (in this study, the absorption spectra) of such perfectly ordered structures by computing these properties based on the assumed molecular packing. This allows us to discard structures which lead to evidently off-target optical properties. In step 3, we perform MD simulations on the remaining structures to assess their stability. At this stage, putting in the atomistic detail of the chromophore packing may lead to tubes which readily distort or disintegrate during the MD simulation. At the same time, the MD sampling leads to structural disorder (*i.e.* deviations from perfectly ordered packing) for the tubes that are stable. The structural disorder translates into disorder in the intermolecular resonance interactions (off-diagonal disorder). In step 4, for stable aggregates, we perform microelectrostatic calculations, including the effect of electronic polarizability, to obtain the influence of the local environment on the molecular excitation energies. This allows us to quantify the energetic, or diagonal, disorder. The diagonal and off-diagonal disorder obtained in steps 3 and 4 are used in step 5, where we calculate the absorption spectrum based on the obtained MD structure, including both types of disorder. This leads to broadening and shifts of absorption peaks compared to the spectrum obtained in step 2. If the resulting spectrum does not agree with the experimental reference spectrum, we use the collected insights as feedback for a new step 1 iteration. If the resulting spectrum agrees well with the experimental one, the validated molecular model is obtained.

Combinations of MD simulations and quantum chemical approaches have been used before to study molecular aggregates. This is true in particular for photosynthetic light-harvesting systems, such as the FMO complex and LH2.^40–44^ Importantly, for these rather small systems, X-ray structures have been determined with atomic-scale resolution, which removes the necessity to find the structure computationally, the challenge we are faced with. MD simulations are then solely used to acquire information about structural fluctuations. For the much bigger chlorosomes of green sulfur bacteria, detailed structural information from direct imaging is lacking, similar to the large synthetic aggregates we are interested in. Very recently, a computational study was reported on chlorosomes using an approach resembling ours.^45^ Complicating factors for the type of aggregates we consider, are that our amphiphilic dye molecules are considerably more challenging from an MD point of view than chlorophyll molecules; the situation is further complicated by the presence of solvated counterions in our systems.

### Description of experimental data on which we rely

The experimental absorption spectrum for C8S3 double-walled aggregates^39^ serves as our reference for judging the validity of the structure. This complicated spectrum shows two sharp low-energy J-bands and several broader higher-energy bands for which the following has been established experimentally: (i) the two sharp peaks, separated by 300 cm^−1^, can be associated with the two separate walls of the aggregate, with the lowest-energy peak deriving from the inner wall (IW) and the upper one from the outer wall (OW).^23^ The two walls are coupled structurally, but do not share collective electronic states responsible for the optical response.^23,46^ (ii) The ratio of the oscillator strengths of both sharp peaks is known from experiment.^23^ (iii) The two sharp low-energy absorption bands are polarized parallel to the tube's axis, while the higher-energy bands also show regions of perpendicular polarization.^23^ (iv) Besides requiring reproduction of these spectral features, we base our modeling on the available experimental structural data. In particular, these are the IW and OW radii, which are 3.2 ± 0.5 nm and 6.5 ± 0.5 nm, respectively, as defined by the positions of the sulfonate groups of the IW and OW obtained from cryo-TEM imaging.^23^ These radii allow for a wall-to-wall distance of 3.3 ± 1.0 nm.

We note that the same (or similar) spectral and structural data have been used as reference points for the phenomenological modeling, performed in ref. 23. The big step forward in the current multiscale approach is that we obtain a true atomistic structure and validate the stability of this structure (and indeed find a packing that is different from the one found phenomenologically). Moreover, using this approach we get microscopic information about structural and energetic disorder.

### Detailed description of the multiscale approach to probe molecular packing

**Fig. 1:**
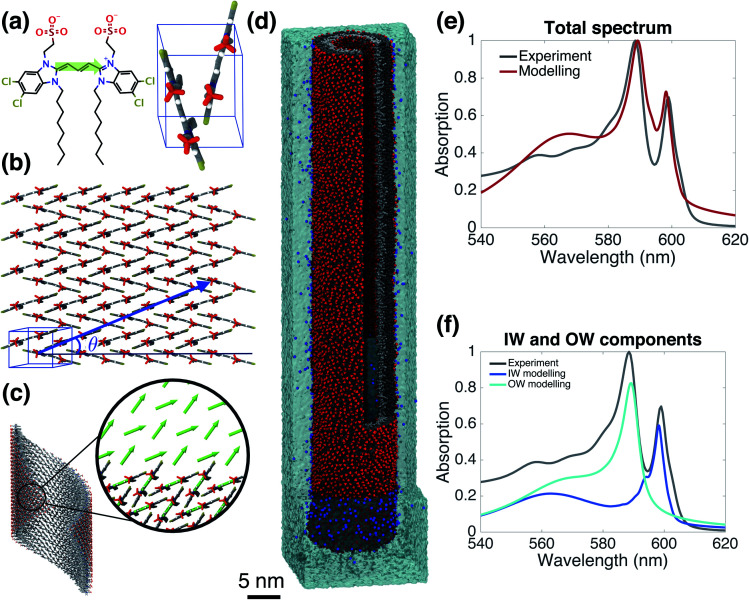
The model system and comparison of modeled and experimental absorption spectra. (a) A C8S3 molecule overlayed with its transition dipole moment (green arrow) and a basic unit cell including two molecules with a herringbone arrangement. (b) Two-dimensional herringbone lattice and rolling vector (blue arrow) with corresponding rolling angle *θ*. (c) Single cylinder obtained from the rolling of the two-dimensional lattice along the rolling vector; in the inset, the arrows represent the transition dipole moments used in the Frenkel exciton Hamiltonian—their positions and orientations are imposed by the molecular structure. (d) A representative snapshot of the double-walled C8S3 nanotube for the optimal (defined as best reproducing the measured absorption spectrum) structure during a 100 ns MD simulation in water; sulfonate groups are in red, carbon atoms in grey (light (dark) grey for inner (outer) wall molecules), water in cyan, and Na^+^ in blue. (e) Absorption spectrum calculated for the modeled system with the optimal structure averaged over a 20 ns MD simulation, compared to the experimental one.^39^ (f) Contributions to the absorption spectrum from the inner and outer walls for the optimal structure.

Starting from the unit cell of a similar molecule^37^ with herringbone formation, we construct a two-dimensional lattice with two C8S3 molecules per unit cell which we then roll onto a cylindrical surface to obtain a tubular structure. In this way, we construct an initial set of ideal—perfectly ordered—single-walled tubes with varying structural parameters: unit cell parameters, density of molecules in the lattice, radius (within the experimental margins), and rolling angle (step 1 of Scheme 1 and Fig. 1a–c).

Next, we select candidates for the IW and the OW based on the comparison of the calculated absorption spectra for this set of ideal single-walled tubes to the experimental spectrum, and preassemble them into double-walled aggregates (up to 100 nm long, ∼7000 C8S3 molecules) (step 2 of Scheme 1). We note that while the cryo-TEM data allow for a wall-to-wall distance of 3.3 ± 1.0 nm, we found that a distance of 2.3–2.7 nm leads to stable structures. Moreover, based on the absorption spectra, we found the rolling angle around 30° to be optimal. The sensitivity to this rolling angle will be discussed further below.

The preassembled double-walled aggregates are used as starting point for MD simulations in an explicit solvent environment, consisting of water and dissolved Na^+^ counter ions (step 3 of Scheme 1). During the MD simulations of 20–100 ns, the modeled systems “acquire” realistic structural disorder, *i.e.* disorder in molecular packing due to the interaction with the solvent and within the aggregate itself (Fig. 1d), and potentially “disintegrate” if the structure turns out to be not stable. Stability is assessed, *inter alia*, by monitoring mass density profiles (see ESI, Section 2.2†).

From the obtained MD snapshots, we estimate how the environment of each chromophore changes the gas-phase monomer excitation energy *via* atomistic microelectrostatic calculations, resulting in a distribution for these solvent shifts, whose standard deviation characterizes the so-called energetic (diagonal) disorder (step 4 of Scheme 1). Note that before doing the expensive calculations in step 4, we do a check by comparing the calculated absorption spectrum in the absence of energetic disorder to the experimental spectrum (we consider peak energies, oscillator strengths, and polarization properties).

Finally, we model the absorption spectrum of the obtained aggregate to validate its structure (step 5 of Scheme 1). For each snapshot, taken every 100 ps along the MD trajectory, we translate the structural information into a Frenkel exciton Hamiltonian that describes the assembly's collective excited states responsible for the optical response; this Hamiltonian accounts for interaction (off-diagonal) disorder arising from orientational and positional disorder. For calculating the intermolecular excitonic couplings, we use the extended dipole model,^47,48^ where the transition dipole is mapped on the polymethine bridge of each C8S3 molecule (Fig. 1a). Furthermore, for each snapshot a random energy shift taken from a Gaussian distribution with mean and width obtained from the above microelectrostatic calculations is added to the gas phase monomer excitation energy for each molecule. By averaging the absorption spectra calculated for each snapshot, we obtain the final spectrum. By comparing this spectrum to the experimental one, structural parameters for the double-walled tube are adjusted and tested by repeating one or more of the above steps, until agreement is good and we conclude that we have found the actual (validated) structure.

### Validated model

Fig. 1e and f gives the calculated spectrum for the “optimal” structure whose spectrum best compares to the experimental one taken from ref. 39. The agreement is very good, not only in the overall spectral shape, but also in essential details: (i) the contributions from the inner and outer walls are correctly assigned, *i.e.* the lowest-energy peak from the inner wall is lower in energy than the one from the outer wall, correctly reproducing the assignment found experimentally.^23^ (ii) The relative oscillator strength of these two peaks is reproduced correctly, as are the polarization directions of the optical transitions: the two lowest-energy absorption bands are polarized parallel to the tube's axis, while higher bands also show regions of perpendicular polarization.^23,49^ (iii) The modeled spectrum reproduces well the experimental linewidths and accounts for structural and energetic disorder. Homogeneous broadening is accounted for phenomenologically following the procedure of previous modeling,^23^ in accordance with expected lifetimes based on experimental observations.^26,50^ In principle, studying the time correlation functions of the various disorder contributions which may be obtained from MD trajectories, allows for a microscopic prediction of the homogeneous linewidths. However, this requires an extensive study that goes beyond the scope of the current paper and will be the topic of a forthcoming publication. The spectrum's absolute position was fixed by using the gas-phase excitation energy of a single C8S3 molecule to be 19 498 cm^−1^; this quantity is unknown experimentally, while quantum theoretical calculations on its absolute value have large error bars.

With the above “optimal” model, for the first time, we are in the position of having a C8S3 aggregate model which not only reproduces the experimental spectrum but also contains atomistic information on the packing of thousands of cyanine dye molecules. This is important, because it allows us to answer open questions concerning the structure and the origin of the aggregate's photophysical properties on a microscopic ground, which hitherto was impossible. In the following sections, we unravel: (1) the magnitude and sources of the energetic and structural disorders; (2) insights into collective properties reflected in the absorption spectrum on the one hand, and the subtle interplay between the density of the molecular packing on the other hand, which leads to: (3) the origin of the splitting of the IW and OW low-energy peaks.

### Probing the effect of molecular disorder

Organic materials generally endure the presence of significant disorder in intermolecular excitonic couplings (structural disorder) and disorder in excitation energies (energetic disorder), which can be detrimental or essential to their functional properties. Disorder affects the delocalized nature of the collective excitations of the molecular aggregate and, consequently, its optical and transport properties. In order to study how disorder impacts the (opto-)electronic properties, it is important to be able to estimate the amount of disorder. Previous models of C8S3 (ref. 23 and 39) do not allow for a first-principles estimate of the amount of structural and energetic disorder due to lack of information on the details and fluctuations in the packing of the aggregated and solvent molecules relative to each other. Our multiscale approach gives direct access to such information.

**Fig. 2:**
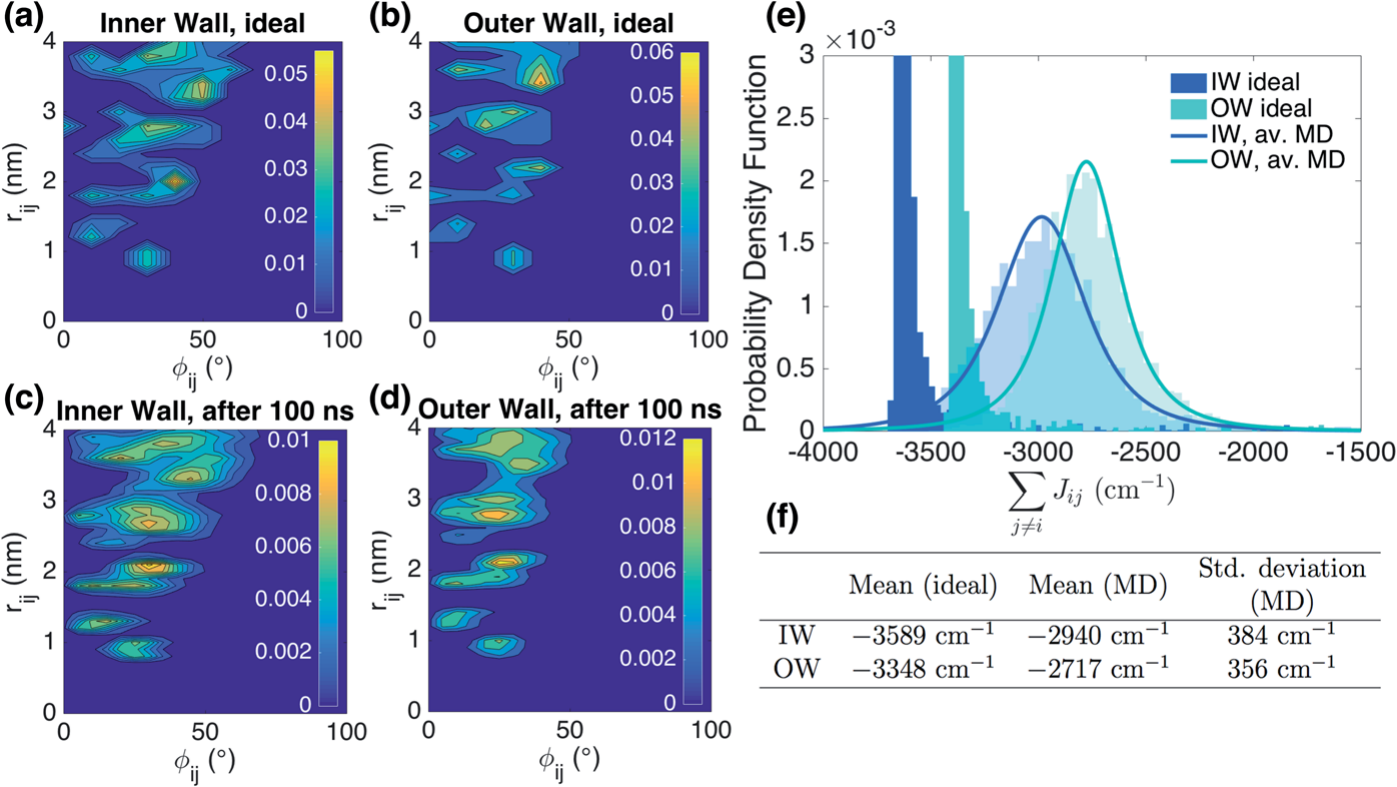
Quantitative presentation of the structural disorder for the optimal system. (a–d) Two-dimensional distributions of the angle between pairs of chromophore transition dipole moments (*ϕ*_*ij*_) and the distance between them (*r*_*ij*_) normalized to the number of pairs, shown separately for both walls. Results for the ideal (starting) structures (a and b) and for the structure obtained after 100 ns of MD simulation (c and d). (e) Histogram of the sum of the intermolecular excitonic interactions per molecule 
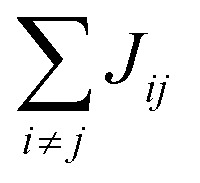
 (ESI eqn (S5)†) for ideal and simulated structures, and (f) table with the corresponding means and standard deviations, averaged over 20 ns.

We start by analyzing the structural disorder due to physical irregularities of the molecular aggregate affecting positions and orientations of the molecules and, therefore, resulting in inhomogeneities in intermolecular interactions. First, we discuss a qualitative picture of the structural disorder, which emerges by plotting the angle between pairs of chromophore transition dipole moments as a function of the distance between them. The obtained maps show how well-defined angle/distance distributions in the ideal (starting) structures (Fig. 2a and b) are smeared out in the disordered structures upon MD simulation, while the overall peak structure is preserved (Fig. 2c and d). The preservation of overall peak structure and limited smearing out of the peaks demonstrates the stability of the obtained structures and implies that the ideal system is still a useful reference, around which the structure fluctuates. Next, the quantitative impact of the structural disorder in the system on the absorption spectrum is obtained from the distribution of the sum of the intermolecular excitonic interactions per molecule, 
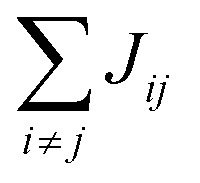
, responsible for the collective excited states of the system (ESI eqn (S5)†). In Fig. 2e and f, we show this distribution averaged over the snapshots of the MD trajectory in comparison to the distribution obtained from the ideal structure. For the ideal structure, this distribution is very narrow with mean values of −3589 cm^−1^ and −3348 cm^−1^ for the IW and OW, respectively. These values are indicative of the red-shifts of the low-energy (parallel) absorption bands of both walls relative to the monomer excitation energies (J-aggregate). For the IW, this shift is larger than for the OW, as a result of the small difference in packing density (see below). The structural disorder acquired in the MD simulation, gives rise to broadening of the distribution and leads to a decrease of the mean value; in the spectra, this leads to broadening and blue-shifts of the spectral peaks relative to the spectra of the starting structures. The width of the distribution and the decrease of the mean value is slightly larger for the IW than for the OW. This effect was seen for all the tubes and can be explained by the fact that the anionic sulfonate groups inside the IW are packed more closely than those outside the OW; the interactions between these groups in the IW thus lead to a larger stress, in turn leading to more structural disorder.

**Fig. 3:**
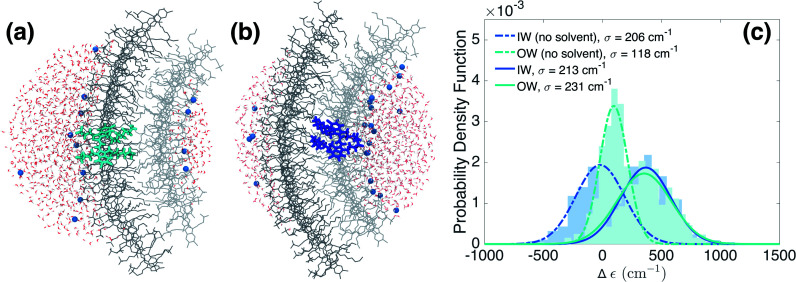
Energetic disorder and energy shifts computed using microelectrostatic calculations. Based on the MD trajectory, microelectrostatic calculations have been performed on clusters of molecules positioned around a central dye molecule residing either in the outer ((a), cyan) or inner ((b), blue) walls. The solvent (water in red and white, and Na^+^ in blue) is treated explicitly. The central molecule's ground and excited state charge distributions interact differently with the environment. The difference between the ground and excited state electrostatic and polarization interaction energies, *i.e.* the solvent shift Δ*ε*, is plotted in (c) for molecules residing in the outer (cyan) and inner (blue) walls. Notably, discarding electrostatic interactions with the solvent, a different Δ*ε* in the two walls is induced by a different electrostatic interaction with the polar heads of the dye molecules as a result of the curvature (dashed lines). This effect cancels out, however, when also accounting for the interactions with the solvent (solid lines).

We now turn to the disorder in molecular excitation energies imposed by the environment. To probe possible differences between molecular excitation energies in both walls as well as disorder in these energies, resulting from the local electrostatic environments around molecules in the IW and OW, we evaluate electrostatic and polarization (or induction) interaction energies between single molecules and their surroundings (Fig. 3a and b) *via* microelectrostatic calculations. Molecules are treated classically and described by atomic point charges (that differ for the molecular ground and excited states) and atomic polarizabilities. The shift in the excitation energy induced by the molecular surroundings is calculated as the difference Δ*ε* = *E*_e_ − *E*_g_ between the interaction energies *E*_e_ and *E*_g_ of the charges and polarizabilities characterizing the molecular excited and ground states, respectively, with the charges and polarizabilities characterizing the surrounding molecules.

Fig. 3c shows the distribution of the computed energy shifts for all the molecules of a 10 nm thick fragment taken from the middle of the aggregate, containing approximately 330 and 450 IW and OW molecules, respectively. As can be seen, molecules in both walls experience very similar electrostatic environments, reflected by the fact that the shift distributions for both walls are practically identical, with mean values around 350 cm^−1^. The standard deviations of the Gaussian functions fitted to these distributions are found to be 213 and 231 cm^−1^ for the IW and OW, respectively, and is a measure of the energetic disorder. This disorder was used when calculating the spectrum in Fig. 1 by adding in the Frenkel exciton Hamiltonian for each C8S3 molecule a random shift relative to the gas-phase excitation energy taken from the corresponding (IW or OW) Gaussian distribution.

### Understanding the energy splitting between the two lowest-energy peaks

Assuming that the underlying lattice is the same for the IW and OW, it is not possible to explain the energy splitting between the lowest-energy optical bands of both walls, polarized parallel to the tube's axis. This is because the energy position of the parallel component of the spectrum of a cylindrical aggregate is essentially independent of both the rolling vector^47^ and the tube's radius.^39^ Moreover, the above calculations show that systematic differences in solvent shifts do not occur and also shifts induced by structural disorder can not explain the energy splitting.

Thus, we are left with the explanation that the energy splitting arises from a slightly different packing density in both walls. A higher density in the IW, as compared to the OW, would ensure stronger excitonic couplings *J*_*ij*_ and therefore a larger red-shift of the lowest-energy absorption peak for the IW. To test this hypothesis, we construct starting aggregate structures assuming different densities of molecules in the two walls. It turns out that enlarging the in-plane unit cell lattice constants for the OW by 2.4% compared to the IW (see ESI, Table S1†) is enough to match the energy splitting in the experimental spectrum. Our MD simulations performed on multiple preassembled structures confirmed that such structures are stable and do not disintegrate. Only because in the above we ruled out relative shifts between both walls induced by surroundings and structural disorder, are we able to conclude on this small difference in density. We believe that this conclusion is solid, because possible errors in the exciton energies that might result from the way we treat solvent effects and intermolecular excitation transfer interactions, are expected to be very similar for the IW and the OW.

### Solvent *vs.* intra-aggregate sources of disorder

Our microelectrostatic calculations exclude the hypothesis of the energy splitting being due to different excitation energies in the IW and OW as a result of different electrostatic environments, used in the phenomenological modeling of ref. 38. It is instructive, however, to disentangle the different contributions to the distributions of Fig. 3c. In particular, removing the solvent (water and counterions) from the microelectrostatic calculations results in a shift of both distributions toward more stabilizing energies as compared to the full calculation (Fig. 3c, dashed lines). A relative shift between the IW and OW then also appears, with the OW Δ*ε* less stabilizing than the IW. Further investigations show that this shift between the two walls is induced by a different electrostatic interaction with the polar heads of the surrounding dye molecules in the aggregate (see ESI Fig. S4b†). This is likely related to the inward *vs.* outward orientation of the polar groups. Interestingly, this relative shift between both walls caused by intra-aggregate interactions, in the end is compensated entirely by an opposite relative shift caused by the interactions of the dye molecules with the solvent. Further investigation shows that the effect of the water is the same for IW and OW molecules, implying that the compensation of the shift between the walls observed without solvent comes from the Na^+^ counterions (ESI Fig. S4a†).

### Sensitivity to the rolling angle

**Fig. 4:**
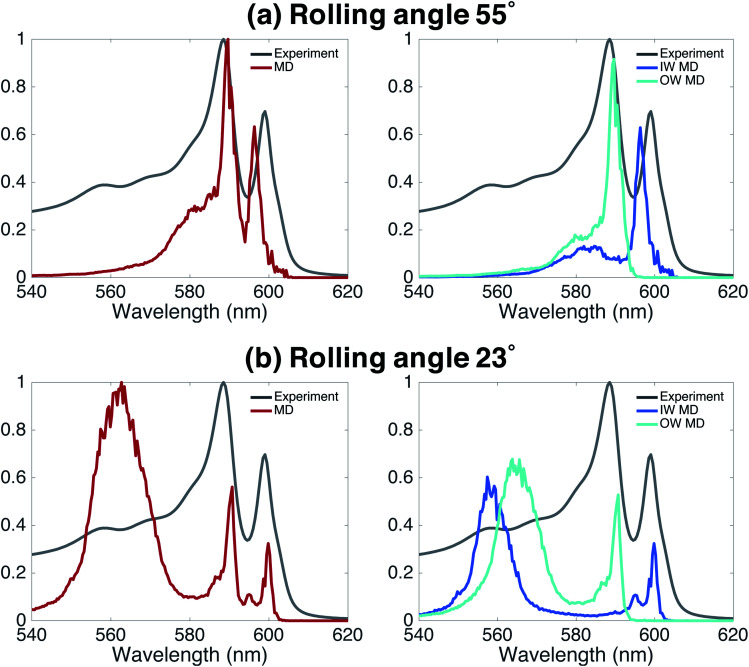
Effect of rolling angle on absorption spectra. The left panels display the total absorption spectra calculated based on an MD trajectory of 20 ns. The right panels show the contributions from both walls. (a) A rolling angle of 55° results in a total lack of oscillator strength in the high-energy region and too much oscillator strength in the two low-energy peaks polarized parallel to the tube's axis. (b) A rolling angle of 23° gives rise to too small an oscillator strength in the parallel polarized bands compared to the perpendicular (higher-energy) bands. In the spectra displayed here, the spectral broadening due to energetic disorder and lifetime were not taken into account.

Finally, we underline the importance of taking into account both molecular dynamics (to assess stability) and exciton modeling (to assess excitonic properties such as the spectrum) to fully be able to determine structural parameters of the model. We do so by looking at the rolling angle, *θ*, of the lattice (Fig. 1b), which strongly influences the optical spectra. It was previously shown that the energy position of the optical band polarized parallel to the axis of tubular assemblies is not sensitive to variations in the rolling angle *θ*.^47^ By contrast, the energy of the optical band polarized perpendicular to the tube's axis strongly depends on *θ*. Moreover, *θ* influences the relative oscillator strength of the parallel and perpendicular polarized bands. We performed a systematic study of structure and absorption properties in order to determine the rolling angle. We found that a rolling angle around 30° ensures a good separation between parallel and perpendicular transitions and gives rise to relative oscillator strengths in agreement with those found in the experiment (Fig. 1e and f). By contrast, Fig. 4 shows examples of calculated spectra of proposed structures that were discarded as candidates. Structures with a rolling angle of 55° (Fig. 4a) result in spectra that lack oscillator strength in the high-energy region (550–570 nm), where the experimental spectrum has considerable intensity.^51^ A rolling angle of 23° (Fig. 4b) results in too small an oscillator strength of the parallel polarized bands compared to the perpendicular ones. Importantly, however, we found that the rolling angle does not influence the stability of the molecular aggregates and order in the molecular packing in the MD simulations (see also ESI Fig. S2 and S3†).

Finding the optimal structure from MD alone, would require a free energy calculation for different rolling angles and radii. However, assessing the free energy differences given by the force field between tubes of different rolling angle and IW and OW dimensions is a major challenge for these types of aggregates, because different arrangements are deep local minima, which is visible in attempted spontaneous self-assembly simulations.^30^ Although the packing of the chromophores is tight, the system as a whole is not a solid. Therefore, we judge that neither thermodynamic integration^52^ along one or more smooth coupling parameters (rolling angle, IW and OW dimensions) nor paths using an Einstein solid as a ref. 53 are feasible (in the latter case a non-interacting system in which all atoms are restrained to their positions is used as common reference, which has been used to calculate the free energy difference of different polymorphs, see *e.g.*ref. 54). Consequently, the rolling angle can not be determined from the MD simulations alone, highlighting the importance of combining several theoretical methods into the multiscale approach used here.

## Conclusions

We have applied a multiscale approach to reveal molecular structure–optical property relationships of a complex self-assembled structure of thousands of molecules, namely the double-walled tubular aggregates of C8S3 dye molecules. By combining (i) molecular dynamics simulations on preassembled structures to test the stability of the nanotubes and to retrieve realistic structural and corresponding interaction disorder, (ii) microelectrostatic calculations to probe different electrostatic environments experienced by the molecules of the system and to retrieve solvent shifts and energetic disorder, (iii) translating aggregate structures obtained from the molecular dynamics simulation into a Frenkel exciton Hamiltonian to model optical signatures, and (iv) using the comparison with experiment to guide modeling and validate the obtained structures, we found optimal structural parameters for the complex molecular packing of these systems. We believe that the obtained structure is the unique solution for the wide family of lattices with herringbone packing considered by us that obeys all experimental (optical and structural) constraints. The absorption spectrum of the proposed optimal molecular structure correctly predicts the optical features of the nanotube: namely the order of the optical bands, their relative oscillator strengths and polarization properties, and the optical bandwidths. We explained the origin of the energy splitting between the inner and outer walls as originating from a slightly different molecular packing in both walls.

The proposed multiscale approach is a powerful tool to obtain understanding of the structural and optical relationships that can be used to reveal design rules for creating new materials. The obtained models with realistic structural parameters and disorder can be further used for detailed studies of the exciton dynamics in these chlorosome mimicking systems. The demonstrated methodology will prove useful for studying the structure and structure–property relationships for other complex supramolecular systems.

## Methods

### Molecular dynamics simulation of preassembled double-walled tubes

A set of preassembled double-walled structures was constructed by independently rolling a two-dimensional lattice, based on the adapted unit cell of a similar molecule,^37^ onto two cylindrical surfaces as described in Section 1 of the ESI.† MD simulations of preselected preassembled double-walled nanotubes were conducted using the Gromacs 5.1.4 simulation package^55,56^ with the GAFF force field.^57^ The relevant force fields were determined in a separate study.^58^ Preassembled systems were solvated in orthogonal boxes of dimensions of approximately 20 × 20 × 130 nm^3^ with periodic boundary conditions using the TIP3P water model.^59^ Moreover, to neutralize the excess negative charge of C8S3 molecules, Na^+^ ions were added. This leads to a system with a total of approximately 4.2 million atoms. Initially, each system was minimized and equilibrated in NVT and NPT conditions followed by the production phase. The production phase was conducted in the NPT ensemble and lasted at least 20 ns or longer. It was found that it is important to initially place the Na^+^ ions in a ratio close to 1/1 near the negatively charged C8S3 molecules, *i.e.* the number of Na^+^ ions near each wall must be close to the number of C8S3 molecules in that wall. Additional information for the MD parameters and the system preparation details are provided in Section 2 of the ESI.†

### Microelectrostatic calculations

The energetic disorder and the relative energy shifts for the IW and OW were computed by means of a microelectrostatic scheme (see ref. 60 and 61 for a recent review). The classical energy expression of the Direct Reaction Field (DRF) approach was used, as implemented in the DRF90 software.^62^ In such a polarizable classical description, molecules are described by atomic point charges and atom-centered isotropic polarizabilities. The system can be thought of as being composed of two subsystems: a central molecule and its surroundings. The energy shift of a single C8S3 molecule was obtained by performing two calculations: one in which the C8S3 molecule is described by the charge distribution of its ground state, and one in which it is described by the charge distribution of its brightest excited state (charge distributions can be downloaded from Figshare^63^). In both calculations, the C8S3 molecules were surrounded by a polarizable surroundings comprising all the C8S3 and solvent (water, Na^+^) molecules within a radius of 3.0 nm from the center of geometry of the C8S3 molecule (see Fig. 3a and b). The surrounding molecules were described by their ground state charge distributions. From these two calculations, interaction energies between the central molecule represented by its ground state (*E*_g_) or excited state (*E*_e_) charge distribution and its surroundings are obtained. The energy shift, Δ*ε*, was then obtained as the difference (*E*_e_ − *E*_g_). We refer to ESI Section 3† for further details on the atomic charges, polarizabilities, radius of the surroundings, and contributions to Δ*ε*.

### Optical modeling

Electronic excited states of the system are described by a Frenkel exciton Hamiltonian^64^ that takes into account dipole–dipole intermolecular excitation transfer (resonance) interactions.^23,48^ The expression for the Hamiltonian is given in eqn (S4) of the ESI.† Vibronic coupling is ignored, because in J-aggregates with strong excitonic coupling (where the exciton band width *W* exceeds the product of the Huang–Rhys factor *λ*^2^ and the vibrational frequency *ω*_vib_) and strong exciton delocalization, vibronic side-bands in the absorption spectrum are strongly suppressed.^65^ This situation applies here: *W* ≈ 6000 cm^−1^, as obtained from our study, while *λ*^2^ ≈ 0.7 (ref. 38) and *ω*_vib_ ≈ 1000 cm^−1^.^38^ The exciton delocalization size in these tubular aggregates generally is known to be very large;^66^ the disorder distributions found in our study lead to delocalization over 450 molecules.^67^

Exciton states are obtained by numerical diagonalization of the Hamiltonian. Homogeneous spectra are obtained from the ideal—cylindrically symmetric—structures assuming the same excitation energies for all molecules. Spectra of the disordered structures are obtained by averaging over the snapshots along the MD trajectory, from which the structural parameters are directly translated into couplings in the Hamiltonian, and energetic disorder is included by adding Gaussian random numbers to the gas-phase molecular excitation energies. The mean value and the width of the Gaussian distribution were estimated by microelectrostatic calculations as described above. For the final spectra, homogeneous broadening was added phenomenologically according to the procedure described in ref. 23. Further details are given in Section 4 of the ESI.†

## Author contributions

J. K. and S. J. M. developed the project. I. P. and A. H. d. V. performed molecular dynamics simulations. A. S. B. performed optical modeling. R. A. performed microelectrostatic calculations. P. C. T. S. provided key insights for the molecular dynamics simulations. A. S. B., I. P., and R. A. analyzed the data under the supervision of T. L. C. J., A. H. d. V., S. J. M., and J. K. A. S. B. and J. K. wrote the manuscript with input from all other authors.

## Conflicts of interest

The authors declare no competing interests.

## Supplementary Material

SC-011-D0SC03110K-s001
