# Supplementary material for: Multiscale modeling of molecular structure and optical properties of complex supramolecular aggregates
Source: Chem Sci. 2020 Oct 1;11(42):11514–24. doi: 10.1039/d0sc03110k (PMC8162738; doi:10.1039/d0sc03110k)
Supplement: SC-011-D0SC03110K-s001 [file SC-011-D0SC03110K-s001.pdf]

Electronic Supplementary Material (ESI) for Chemical Science.
This journal is © The Royal Society of Chemistry 2020

Supplementary Information for: Multiscale Modeling of Molecular Structure and Optical Properties of Complex Supramolecular Aggregates

Anna S. Bondarenko,[†] Ilias Patmanidis,[†,‡] Riccardo Alessandri,[†,‡] Paulo C.T. Souza,[†,‡] Thomas L.C. Jansen,[†] Alex H. de Vries,[†,‡] Siewert J. Marrink,[*,†,‡] and Jasper Knoester[*,†]

†*University of Groningen, Zernike Institute for Advanced Materials, Groningen, The Netherlands*

‡*University of Groningen, Groningen Biomolecular Sciences and Biotechnology Institute, Groningen, The Netherlands*

E-mail: s.j.marrink@rug.nl; j.knoester@rug.nl

Contents

1 Obtaining the Preassembled Structures **3**

1.1 Structural restraints 3

1.2 Rolling of the herringbone lattice on the cylindrical surface 4

2 MD Simulations on the Preassembled Structures **5**

2.1 MD protocol 5

2.2 Stability of the tubes 7

3 Probing the Energetic Disorder **9**

3.1 Microelectrostatic calculations 9

3.2 Electronic structure calculations 13

4 Absorption Spectra Calculation **16**

References **18**

1 Obtaining the Preassembled Structures

1.1 Structural restraints

The available experimental data and previous modeling based on phenomenological studies allow us to set restraints for the construction of the initial set of tubes. For the tube's radius, we use values obtained from the cryo-TEM scan profile,[1] which are equal to 3.2±0.5 nm and 6.5±0.5 nm for the sulfonate groups of the inner and outer walls, respectively. For all the tubes, we find a wall-to-wall distance of 2.3-2.7 nm taken from the sulfonate groups to be optimal. This wall-to-wall distance agrees with studies on small-scale nanotubes and bilayers.[2] Moreover, we adhere to the herringbone arrangement proposed for the underlying molecular packing in this aggregate.[1] Using phenomenological modeling, a herringbone lattice with two molecules per unit cell results in a Davydov splitting of the optical bands, which explains well the complex optical spectrum of the C8S3 aggregate[1] and the optical changes for a modified version of the aggregate with an increased radius.[3] By contrast, a brickwork or staircase lattice with one molecule per unit cell cannot explain the multitude of peaks in the absorption spectra of these aggregates.[1] Thus, as a starting point we take a unit cell with the herringbone arrangement for a similar molecule—namely, TTBC—obtained crystallographically.[4,5] The unit cell has the following lattice parameters:[5] $a = 22.547$ Å, $b = 11.036$ Å, $c = 13.375$ Å, $\alpha = 90°$, $\beta = 107.48°$ and $\gamma = 90°$. Keeping the arrangement of the cores of the chromophores unchanged and replacing the side chains of TTBC for those of C8S3, we obtain a starting unit cell for our modeling (Figure S1a). Preliminary analysis of the arrangement of the C8S3 monomers showed that for optimal arrangement, an increase of the length of the unit cell (parameter b) was necessary. Consequently, we used $b = 12.036$ Å and one of the monomers was translated by 1.0 Å in the b-direction to accommodate the change in the unit cell.

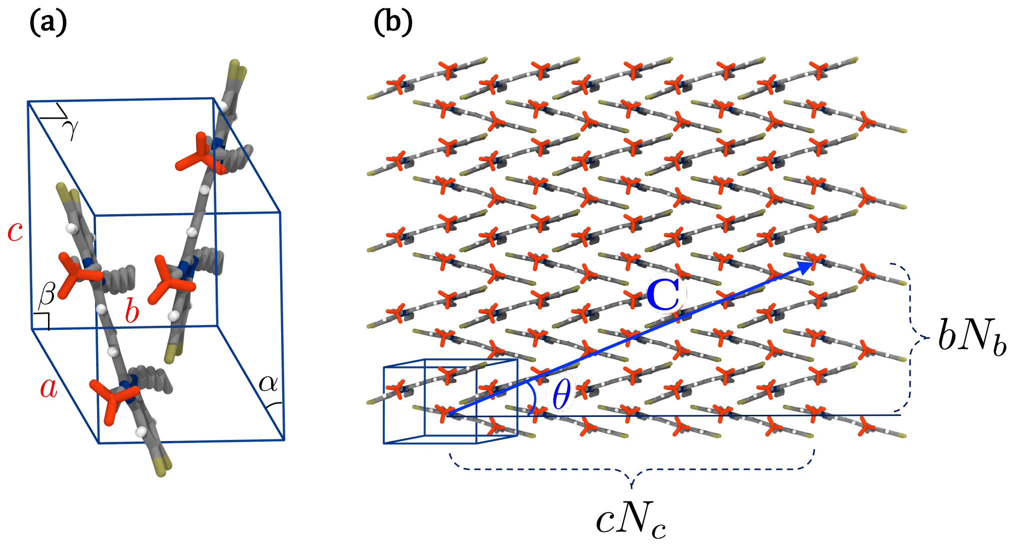

Figure S1: **Construction of the herringbone lattice.** a) Unit cell with two molecules. b) Herringbone lattice.

1.2 Rolling of the herringbone lattice on the cylindrical surface

From the modified unit cell we construct two-dimensional herringbone lattices (Figure S1b). Briefly, we used an in-house python script to take the atoms in the unit cell constructed as described in the previous section to eventually build a double-walled structure. To create a two-dimensional lattice, the unit cell is replicated along a and b directions N_a and N_b times, respectively. We wrap this lattice along the rolling vector $\mathbf{C}$, by connecting its beginning and end points, onto a cylinder resulting in a tubular structure (Figure 1c of the main text). The circumference of this tube is equal to the length of the rolling vector $\mathbf{C}$, and is related to the tube's radius R via the relation $|\mathbf{C}| = 2\pi R$. The value of R, taken within the experimental errors,[1] therefore, restricts the range for the values of the length of the rolling vector. We construct a set of tubes with varying rolling angles (θ), as this angle is not known from the experiment. The values of θ and R acquire discrete values restricted by the dimensions of the unit cell and are defined by the the following equations:

$$R = \frac{1}{2\pi}\sqrt{(bN_b)^2 + (cN_c)^2} \tag{S1}$$

$$\cos\theta = \frac{cN_c}{2\pi R} \tag{S2}$$

From the obtained single-walled tubes, we construct a set of double-walled tubes (making sure the thickness is equal to 1.7 ± 0.2 nm) with length $75 - 100$ nm, resulting in aggregates containing about $5,400 - 7,000$ C8S3 monomers. The rolling angle is varied between 20° and 55°.

Structural parameters of the inner and outer walls of the model tube used in Figure 1 of the main text are summarized in Table S1.

Table S1: Structural parameters used for the model in Figure 1 of the main text.

	a, Å	b, Å	c, Å	R, nm	θ, °
IW	22.547	12.036	13.375	3.724	31.0
OW	22.547	12.324	13.696	5.442	32.7

2 MD Simulations on the Preassembled Structures

2.1 MD protocol

MD simulations of preselected preassembled double-walled nanotubes were performed using the Gromacs 5.1.4 simulation package[6,7] with the GAFF force field.[8] To neutralize the excess negative charge of C8S3 molecules, Na^+ ions were added. A random placement of ions in the simulation box (using, for example, the Gromacs tool `gmx solvate`) distributes ions homogeneously across the solvent phase; however, such a homogeneous distribution results in a local charge imbalance at the negatively charged surfaces of the walls. This local imbalance is particularly strong in the case of the IW due to the considerably smaller solvent volume inside the tube compared to the one outside the tube. This local charge imbalance leads to considerable stress in the tube at the beginning of the MD simulations, which causes defects in the initial packing. Given the high computational cost of these simulations, such

defects cannot be restored during 100 ns. So, the best strategy is to start the MD with ion shells already near to the nanotube surfaces, as expected for such highly charged structures, and as observed in converged simulations of small-scale nanotubes and bilayers.[9] Thus, Na^+ ions were distributed within cylinders of space, one for the ions neutralizing the IW, one for the ions neutralizing the OW, with a ratio of Na^+/C8S3 close to 1/1 for each wall. The sizes of the cylinders were chosen to ensure that the Na^+ ions were near the C8S3 head groups. Similar ion placement approaches are used in MD simulations of other highly charged systems as nucleic acids[10] and certain bilayers.[11] After counter ion placement, the systems were solvated (by using the Gromacs tool `gmx solvate`) in orthogonal boxes of dimensions of approximately $20 \times 20 \times 130$ nm^3 with periodic boundary conditions using the TIP3P water model.[12] This leads to a system with a total of approximately 4.2 million atoms. Note that the nanotube is not periodic along the longitudinal direction, i.e. its ends are not connected.

After solvation, the systems were minimized with the Steepest Descent algorithm for 1000 steps and subsequently equilibrated in the NVT and NPT ensembles (with a time step of 1 fs) for 10 ps and 5 ns, respectively. During the equilibration, the aromatic core of the C8S3 monomers was restrained using harmonic potentials with a force constant of 1000 kJ mol^{-1} nm^{-2}. In this way, the side chains, ions, and the water molecules were able to relax before the production phase. For the actual MD simulation, carried out in the NPT ensemble, the time step for integration of the equations of motion was set to 2 fs and snapshots were saved every 100 ps. To prevent the aggregates from crossing the periodic boundary conditions, the systems were translated and rotated to remove the center of mass motion of the nanotube every 100 steps. Production simulations lasted at least 20 ns for each simulated tube structure.

For all MD simulations, the temperature was set to 300 K and kept fixed by using the V-rescale algorithm[13] with a coupling constant of 0.1 ps. The Berendsen barostat[14] was used to maintain standard pressure in an isotropic pressure bath with a time constant of

1 ps and compressibility of 4.5 10^{-5} bar^{-1}. Electrostatic and van der Waals interactions were calculated by using the Verlet cut-off scheme with a 1.4 nm cut-off. The long range interactions were calculated with the reaction field method.[15]

2.2 Stability of the tubes

Snapshots of the tubes with two different angles, 23° and 55°, after a 100 ns MD simulation are shown in Figure S2. As can be qualitatively seen, both tubes are stable after 100 ns. This implies that MD simulation alone cannot resolve the rolling angle for these complex supramolecular aggregates.

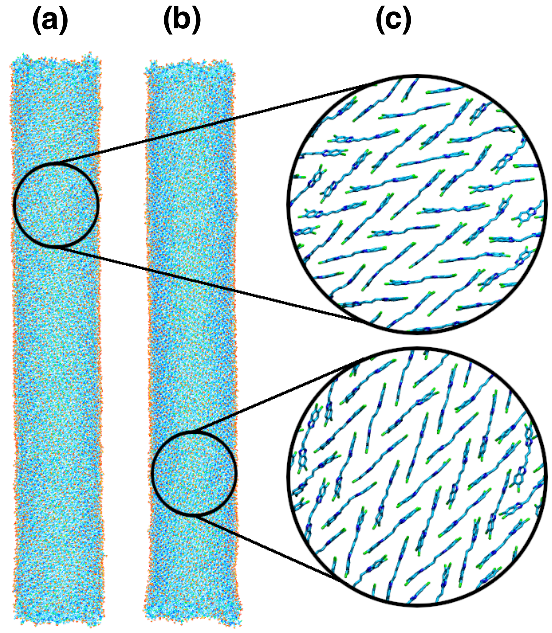

Figure S2: Final MD snapshots of the double-walled tubes after 100 ns. Tubes with rolling angle **(a)** $\theta = 23°$ and **(b)** $\theta = 55°$ are shown. **(c)** Close up of the arrangement of the C8S3 aromatic cores in the two different tubes for the 100 ns frame. Only the aromatic core of the C8S3 molecules (excluding hydrogens) is shown for clarity.

The stability of the tubes was measured by generating mass density plots of the sulfur atoms in a direction perpendicular to the long axis of the tubes along the trajectories. The sulfur density curves have a distinct pattern when the tubular formation is maintained

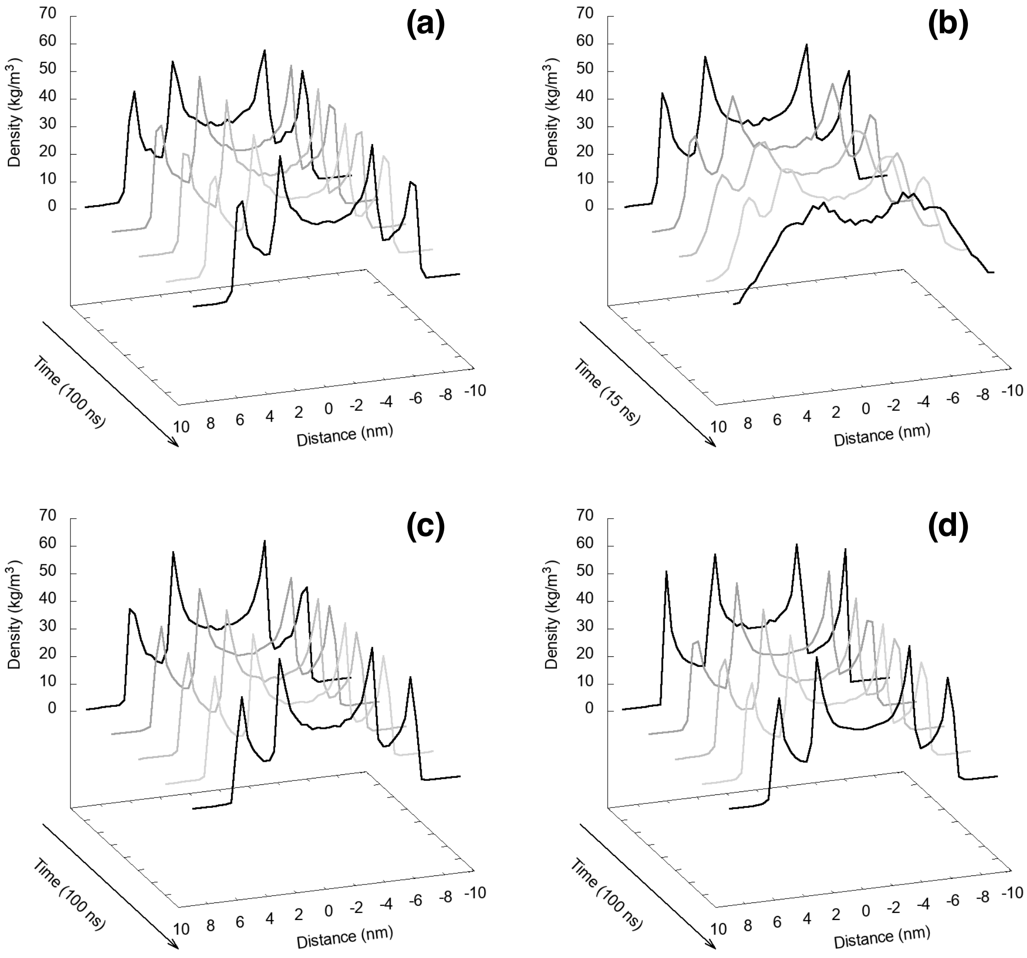

Figure S3: Mass density profile of the double-walled tubes as a function of simulation time. (a) A stable and ordered tube after 100 ns—the absorption spectrum given by this structure is the one reported in Figure 1d of the main text. (b) A tube with a high level of disorder after 15 ns; the simulation was terminated due to the tube's disintegration after 15 ns. Tubes with (c) $\theta = 23°$ and (d) $\theta = 55°$ (Figure 4 of the main text) both being stable along a 100 ns trajectory. In all cases, the profiles were centralized and symmetrized along an axis perpendicular to the tube's axis.

(Figure S3a). The pattern broadens or is even completely lost when the tubes become disordered or they start to fall apart. An example of such disrupted density profile is shown on Figure S3b, where a tube with small interwall thickness and poor Na^+ ion equillibration disintegrates within 15 ns.

3 Probing the Energetic Disorder

3.1 Microelectrostatic calculations

General details. The energetic disorder and the relative energy shifts for the IW and OW were computed by means of a microelectrostatic, or induced dipole, scheme.[16] We employed the classical energy expression of the Direct (or Discrete) Reaction Field (DRF) approach as implemented in the DRF90 software.[17] In such a polarizable classical description, molecules are described by atomic point charges and atom-centered isotropic polarizabilities (see below for details on the charges and polarizabilities used). Polarizabilities are described according to Thole's method for interacting polarizabilities,[18] which avoids numerical instabilities by employing a distance-dependent damping function.

Definition of the energy shift in the excitation energy. The interaction of a C8S3 molecule with its environment generally will be different for its ground and excited states. This leads to a shift in its excitation energy away from the gas phase value (note that we are considering vertical excitations—i.e. no structural rearrangement takes place upon excitation—as those determine the absorption spectrum). We can think of the system as composed of two subsystems: a central molecule and its surrounding (Figure 3a,b of the main text). We represent the ground and excited states of the central molecule with a different set of charges (see Section 3.2). This means that what can change is 1) the electrostatic interaction energy, i.e. the (static) Coulomb interaction between the charges on the central molecules and the surrounding charges, and 2) the polarization interaction energy due to the mutual polarization of the central molecule and its surroundings. We compute

the *electrostatic* ($E^{(\mathrm{el})}$) and *polarization* (or *induction*, $E^{(\mathrm{pol})}$) interaction energies between a central molecule and its surrounding for both the ground state (E_{gs}) and the (brightest) excited state (E_{es}) of the central molecule. The difference

$$E_{\mathrm{es}}^{(\mathrm{tot})} - E_{\mathrm{gs}}^{(\mathrm{tot})} = (E_{\mathrm{es}}^{(\mathrm{pol})} + E_{\mathrm{es}}^{(\mathrm{el})}) - (E_{\mathrm{gs}}^{(\mathrm{pol})} + E_{\mathrm{gs}}^{(\mathrm{el})}) = \Delta\epsilon \tag{S3}$$

yields the shift ($\Delta\epsilon$) to be applied to the gas phase excitation energy.

Input parameters. Calculations are performed with DRF90—the program is available at http://www.marcelswart.eu/drf90/index.html—the classical implementation of the DRF method.[17] The input requires the following parameters: *i)* the atomic coordinates, which are obtained from the MD simulation; *ii)* the atomic charges for the ground and excited states, which are obtained from quantum chemical calculations as described in Section 3.2 below; *iii)* the atomic polarizabilities, which are taken from the standard DRF set of (effective) atomic isotropic polarizabilities, that is a set of atomic polarizabilities parametrized on the basis of a large set of experimental molecular polarisabilities;[17,19] *iv)* the radius of the region surrounding the central molecule, which was set to 30 Å for all the production calculations (see also below for a discussion of the radius of the surrounding region).

In the calculations, the system is considered to be divided in two subsystems: the central molecule and its surroundings. A slab of a C8S3 nanotube of about 10 nm (from the middle) is considered. This comprises a total of approximately 330 and 450 "central" molecules in the inner and outer walls, respectively. Selection of the slab, selection of the surroundings of each central molecule, and preparation of DRF90 inputs are done via a Python script which makes extensive use of the `MDAnalysis`[20,21] library. The surrounding region is spherical, centered at the centre of geometry of the central molecule (see Figure 3a,b of the main text). The effect of the radius of the surrounding region is investigated in Figure S5 and associated discussion. For each "central" molecule, two inputs are generated, with the central molecule bearing either the charges of the ground state or of the excited state. Molecules in the

surrounding are described in both cases by the ground state charge distribution.

Contributions to the energy shift. Figure 3c of the main text shows a blue-shift of the two distribution upon solvation, but the question remains open whether the shift is caused by the water molecules or the Na^+ ions. To investigate this, we carried out additional calculations where the electrostatic interaction with the water was accounted for, but not with the Na^+ ions. Figure S4a shows the distribution of energy shifts for the same snapshot without any solvent, with water, and with the full solvent configuration, i.e. water and Na^+ ions. The results show that the effect of the water is the same for IW and OW molecules and that the compensation of the shift between the walls observed without solvent comes from the Na^+ counterions.

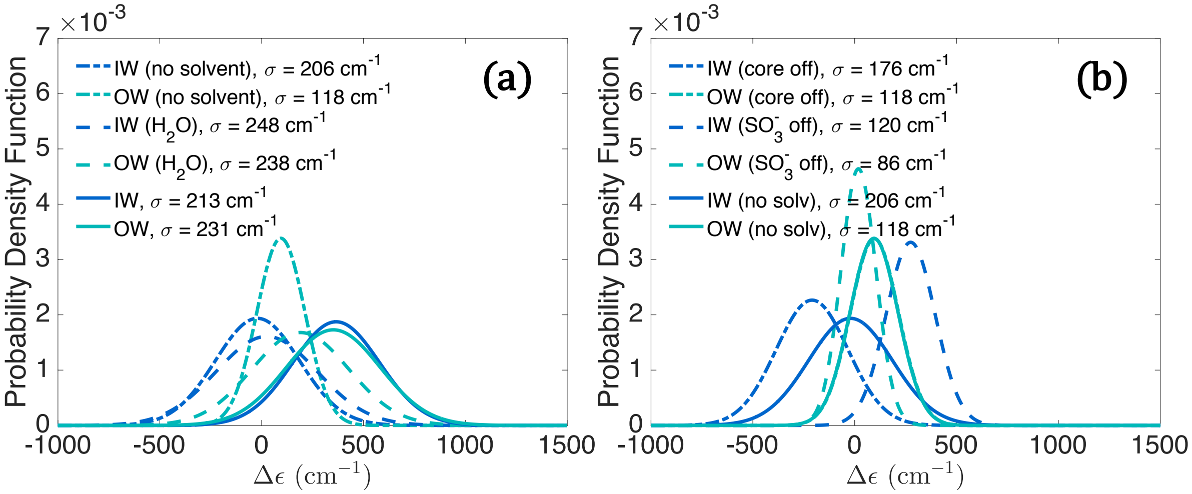

Figure S4: **Contributions to the energy shifts. (a)** Energy shift distributions without any solvent (dashdotted line), with water (dashed), with both water and Na^+ ions (solid). **(b)** Distributions obtained excluding the solvent and when switching off the charges of the aromatic cores (dashdotted line), or of the polar head groups (dashed); the full calculation (excluding the solvent) is also shown (solid). Note that the "OW (core off)" curve is not clearly visible as it overlaps strongly with the "OW (no solv)" one.

It remains to be seen what causes the initial shift which is present when excluding the water and ions. To investigate this, the following calculations were performed: one where the charges on the aromatic core of all but the central molecule were set to zero ("core off"), and and one where the same was done for the polar head groups ("SO_3^- off"). Figure S4b shows that when charges on the polar head groups of the molecules in the surrounding are set to

zero, the relative shift of the IW and OW distributions is inverted. Thus, the stabilization of the IW excitation energies relative to the OW ones in the absence of the solvent is induced by an electrostatic interaction with the polar heads of the C8S3 molecules.

Convergence of microelectrostatic calculations. Figure S5a shows how the distribution and its Gaussian fit change upon varying the number of molecules considered in the calculations. It can be seen that already at 100 molecules the obtained Gaussian distribution is qualitatively converged. In particular, we note how the standard deviation, σ, is very robust and reasonably converged already at 100 molecules. However, the mean value of the distribution, $\overline{\Delta\epsilon}$, is found to be very sensitive to little variations in the distribution, as can be seen from Figure S5a where practically overlapping fits give rise to changes in $\overline{\Delta\epsilon}$ by 10-20 cm^{-1}. All the production calculations consider at least 330 molecules per distribution.

Figure S5b shows the effect of the radius of the surrounding region, varied from 20 to 30 Å. The distributions are very similar in all cases, and particularly between 25 and 30 Å. Again the σ of the distributions converge quickly, while $\overline{\Delta\epsilon}$ is again found to be more sensitive. All production calculations have been run with a radius of 30 Å.

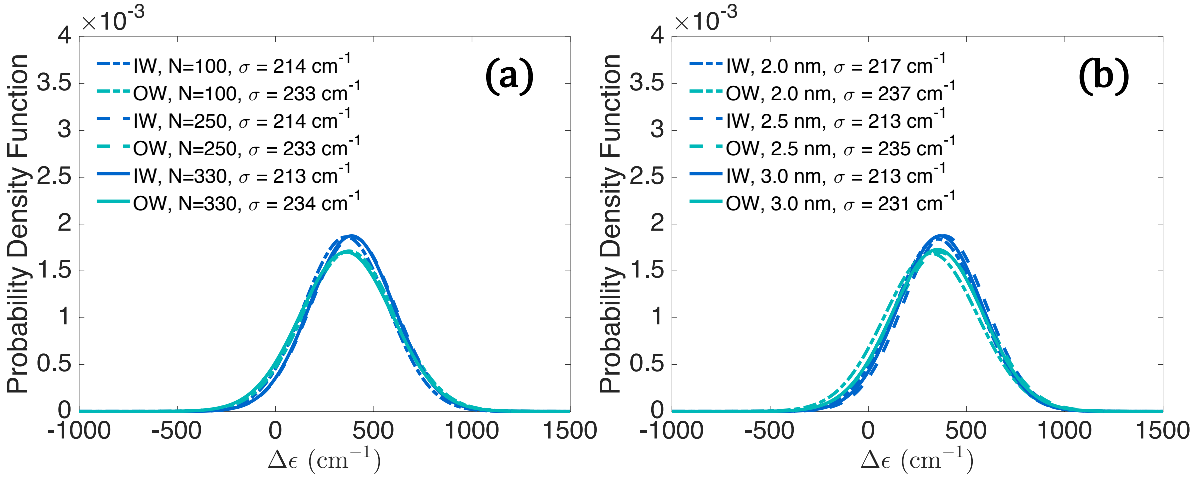

Figure S5: **Convergence of the Gaussian parameters extracted from the microelectrostatic calculations.** The convergence is shown as a function of **(a)** the number of molecules per wall for which the energy shift is computed and **(b)** the size of the surrounding region around the central molecule.

3.2 Electronic structure calculations

The only input parameters required by DRF90 not readily available are the atomic charges of the ground and excited states. Charges which reproduce the electrostatic potential and the dipole moment of the molecule have been computed via the CHELPG[22] scheme as implemented in Gaussian.[23] This is done for both the ground state and the (vertical) excited state with the highest oscillator strength, as obtained via ωB97XD[24]/6-31G(d,p)[25] density functional theory (DFT) and time-dependent DFT (TDDFT)[26] calculations, respectively. Further details on the TDDFT calculations and excited state analysis are reported in Table S2 and discussed below.

Excited state calculations. Table S2 collects the results for the TDDFT calculations in gas phase. The state-of-the-art ωB97XD density functional, containing a long-range correction for the self-interaction error and a dispersion correction, is compared to results obtained with the B3LYP functional. Results obtained with ωB97XD show that the brightest excited state, i.e. the excited state with the largest oscillator strength, is the lowest singlet excited state.

Table S2: Results on the brightest excited state of C8S3 at the TDDFT level. The geometry was optimized at the B3LYP/6-31G(d,p) DFT level. The basis set employed for all the TDDFT calculations is 6-31G(d,p). μ is the transition dipole moment.

DFT functional	Bright State	Orbitals	Energy (nm)	Energy (cm^{-1})	μ^2 (au)	μ (D)	f. osc.
B3LYP	S3	HOMO−2→LUMO	434.7	23,002	22.2741	12.0	1.56
ωB97XD	S1	HOMO→LUMO<br>HOMO−2→LUMO	407.5	24,539	23.0113	12.2	1.71

Atomic charge distributions. CHELPG charge distributions reproducing the electrostatic potential and dipole moment have been obtained for the ground, $\{q^i_{gs}\}$, and brightest excited state, $\{q^i_{es}\}$, at the ωB97XD/6-31G(d,p) level. The difference between these two set of charges, $\{q^i_{es} - q^i_{gs}\}$, is computed and plotted on the optimized molecular structure of C8S3 in Figure S6. As expected,[3] the excitation involves rearrangements of the electron density along the bridge connecting the two benzimidazole rings.

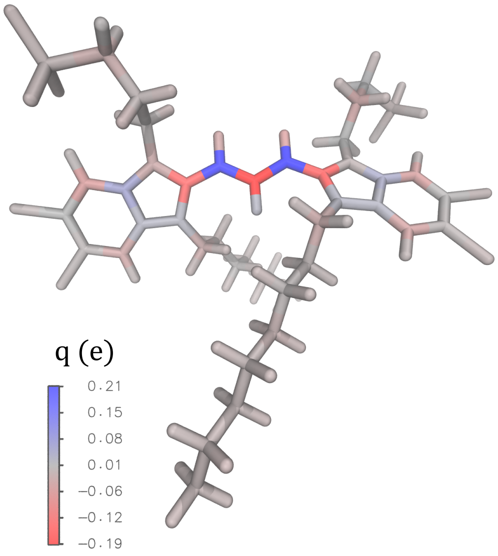

Figure S6: **Difference in CHELPG charge distribution between the brightest excited state and the ground state.** Ground and excited state charge distributions have been obtained at the ωB97XD/6-31G(d,p) (TD)DFT level via the CHELPG scheme. The charge shown on the i-th atom is obtained as $q^i_{es} - q^i_{gs}$, where q^i_{es} and q^i_{gs} indicate the charges on the i-th atom obtained by fitting the electrostatic potential around the molecule given by the density of the (brightest) excited state and of the ground state, respectively. q^i_{es}, q^i_{gs}, and $q^i_{es} - q^i_{gs}$ charges are also reported for the aromatic core and polar chains in Table S3.

Table S3: CHELPG wB97XD/6-31G(d,p) charges for the ground, q^i_{gs}, and brightest excite state, q^i_{es}, and their difference, $q^i_{es} - q^i_{gs}$. Charges for hydrogen atoms, as well as for the hydrophobic tails, are not shown, as their $q^i_{es} - q^i_{gs}$ is less than 0.01 in all cases. All charges can be downloaded at Ref. 27.

Aromatic core	q^i_{gs}	q^i_{es}	$q^i_{es} - q^i_{gs}$
C	0.252958	0.061150	−0.1918
C	−0.543347	−0.329100	0.2142
C	−0.526076	−0.323041	0.2030
C	0.463422	0.326516	−0.1369
C	0.413340	0.294682	−0.1187
N	−0.345175	−0.345127	0.0000
N	−0.293395	−0.312631	−0.0192
N	−0.250184	−0.227282	0.0229
N	−0.249345	−0.228117	0.0212
C	0.186659	0.224476	0.0378
C	0.174878	0.225876	0.0510
C	0.084672	0.107290	0.0226
C	0.102074	0.125245	0.0232
C	−0.145171	−0.182258	−0.0371
C	−0.143487	−0.183603	−0.0401
C	−0.206432	−0.233804	−0.0274
C	−0.214244	−0.238771	−0.0245
C	0.067202	0.076042	0.0088
C	0.048615	0.059744	0.0111
C	0.058889	0.064617	0.0057
C	0.070711	0.076500	0.0058
Cl	−0.081273	−0.081403	−0.0001
Cl	−0.119581	−0.119264	0.0003
Cl	−0.081959	−0.078938	0.0030
Cl	−0.119078	−0.115494	0.0036
Polar chains			
C	0.186200	0.197853	0.0117
C	0.132649	0.138415	0.0058
C	0.098364	0.068027	−0.0303
C	0.181582	0.184990	0.0034
C	−0.277549	−0.263714	0.0138
C	−0.302521	−0.314697	−0.0122
S	1.044482	1.041970	−0.0025
S	1.057744	1.059741	0.0020
O	−0.601670	−0.602064	−0.0004
O	−0.606641	−0.605698	0.0009
O	−0.628265	−0.625947	0.0023
O	−0.644372	−0.643439	0.0009
O	−0.632288	−0.630268	0.0020
O	−0.623714	−0.623384	0.0003
C	0.153515	0.137125	−0.0164
C	0.144272	0.124634	−0.0196

4 Absorption Spectra Calculation

The excitonic systems of inner and outer walls are assumed decoupled.[1] We use a Frenkel exciton Hamiltonian to describe the collective excited states of the excitonic system of either of the walls:

$$H = \sum_{i=1}^{N} (\epsilon_0 + \Delta\epsilon_i) \left|i\right\rangle \left\langle i\right| + \sum_{i \neq j}^{N} J_{ij} \left|i\right\rangle \left\langle j\right|, \tag{S4}$$

where ϵ_0 is a molecular gas-phase excitation energy; $\Delta\epsilon_i$ is the fluctuation of the gas-phase excitation energy on molecule i obtained from a Gaussian distribution of width σ, where the value of σ is estimated from the microelectrostatic calculations; $|i\rangle$ is the state where only molecule i is excited and all other molecules are in the ground state. We use $\epsilon_0 = 19,498$ cm^{-1}. J_{ij} is the intermolecular resonance (excitation transfer) interaction between the molecules i and j.

To calculate J_{ij}, we use the extended transition dipole model,[28,29] where the transition dipole moment of the molecule is considered as a dipole of length l with two point charges $+q$ and $-q$ such that the magnitude and orientation of the relevant molecular transition dipole moment is reproduced. In this approximation, the transfer interaction J_{ij} is explicitly given by:

$$J_{ij} = A\frac{\mu^2}{l^2}\left[\frac{1}{r_{ij}^{++}} - \frac{1}{r_{ij}^{+-}} - \frac{1}{r_{ij}^{-+}} + \frac{1}{r_{ij}^{--}}\right], \tag{S5}$$

with

$$\begin{aligned} r_{ij}^{\pm\pm} &= |\mathbf{r}_{ij} \pm l\frac{(\hat{\mathbf{e}}_i - \hat{\mathbf{e}}_j)}{2}|, \\ r_{ij}^{-+} &= |\mathbf{r}_{ij} - l\frac{(\hat{\mathbf{e}}_i + \hat{\mathbf{e}}_j)}{2}|, \\ r_{ij}^{+-} &= |\mathbf{r}_{ij} + l\frac{(\hat{\mathbf{e}}_i + \hat{\mathbf{e}}_j)}{2}|, \end{aligned} \tag{S6}$$

where the transition dipole moment is related to the charge q and dipole length l ($\mu = ql$), and $\mathbf{r}_{ij} = \mathbf{r}_i - \mathbf{r}_j$. We use the previously reported values[29] $q = 0.34e$ and $l = 0.7$ nm for the point charges and length that give rise to the magnitude of the transition dipole moment of 11.4 D. The molecular position vector $\mathbf{r}_i$ and unit vector $\hat{\mathbf{e}}_i$ that represents the direction

of the transition dipole moment of molecule i, $\boldsymbol{\mu}_i = \mu\hat{\mathbf{e}}_i$, are obtained from the structure from each saved snapshot of the MD trajectory, where $\hat{\mathbf{e}}_i$ is the unit vector between the carbon atoms of the imidazole rings of the C8S3 molecule (Figure 1a of the main text), and r_i is the coordinate of the carbon atom of one of the imidazole rings. The constant $A = 5.04$ cm^{-1} nm^3 Debye^{-2} comes from a conversion of units allowing one to express transition dipole moments, distances, and energies in Debye, nm, and cm^{-1}, respectively.[29]

Linear absorption spectrum. After constructing the Hamiltonian of eq. S4, and numerically diagonalizing it, we obtain the exciton states:

$$|q\rangle = \sum_i \varphi_{qi} |i\rangle , \tag{S7}$$

where φ_{qi} denotes the amplitude of the qth state on molecule i. The corresponding eigenvalue of the state is the energy E_q. This calculation is repeated for every snapshot of the MD simulation. For each snapshot, the molecular energy shifts are taken randomly for each molecule from a Gaussian distribution.

The general form of the linear absorption spectrum for an isotropic solution, obtained from the Fermi golden rule,[30] is given by

$$A(\omega) = \left\langle\left\langle \sum_q O_q \delta(\omega - E_q) \right\rangle\right\rangle, \tag{S8}$$

with the oscillator strength of the state q given by

$$O_q = \sum_{i,j} \varphi_{qi}\varphi_{qj}^* \langle (\boldsymbol{\mu}_i \cdot \mathbf{e})(\boldsymbol{\mu}_j \cdot \mathbf{e}) \rangle. \tag{S9}$$

The double angular brackets $\langle\langle ... \rangle\rangle$ imply an average over snapshots from the MD trajectory, together with the single brackets $\langle ... \rangle$ representing an average over the orientations of the cylinder relative to the electric polarization vector $\mathbf{e}$ of the incident linearly polarized electromagnetic wave. The averaged spectrum captures instantaneous fluctuations of the ex-

citation transfer interactions J_{ij} due to structural as well as energetic disorder and results in broadening of the modeled spectrum. For the Figure 1 of the main text, the calculated transitions are additionally broadened with Lorentzian lineshapes to facilitate the comparison with the experimental spectrum. For the lowest energy Davydov split optical parallel and perpendicular polarized bands of the inner wall, a homogeneous broadening with a full width at half maximum of 40 cm^{-1} and 1000 cm^{-1}, respectively, was used. For the outer wall, we use 110 cm^{-1} and 1000 cm^{-1}, respectively for the lowest optical parallel and perpendicular polarized bands.

References

(1) Eisele, D. M.; Cone, C. W.; Bloemsma, E. A.; Vlaming, S. M.; van der Kwaak, C. G. F.; Silbey, R. J.; Bawendi, M. G.; Knoester, J.; Rabe, J. P.; Vanden Bout, D. A. Utilizing Redox-Chemistry to Elucidate the Nature of Exciton Transitions in Supramolecular Dye Nanotubes. *Nat. Chem.* **2012**, *4*, 655.

(2) Patmanidis, I.; de Vries, A. H.; Wassenaar, T. A.; Wang, W.; Portale, G.; Marrink, S.-J. Structural Characterization of Supramolecular Hollow Nanotubes with Atomistic Simulations and SAXS. **2020**, 10.26434/chemrxiv.12871379.v1.

(3) Kriete, B.; Bondarenko, A. S.; Jumde, V. R.; Franken, L. E.; Minnaard, A. J.; Jansen, T. L. C.; Knoester, J.; Pshenichnikov, M. S. Steering Self-Assembly of Amphiphilic Molecular Nanostructures via Halogen Exchange. *J. Phys. Chem. Lett.* **2017**, *8*, 2895–2901.

(4) Smith, D. L.; Luss, H. R. The crystal structures of two solvates of 5, 5', 6, 6'-tetrachloro-l, 1', 3, 3'-tetraethylbenzimidazolocarbocyanine iodide. *Acta Crystallogr. B* **1972**, *28*, 2793–2806.

(5) Friedl, C.; Renger, T.; Berlepsch, H. v.; Ludwig, K.; Schmidt am Busch, M.; Megow, J. Structure Prediction of Self-Assembled Dye Aggregates from Cryogenic Transmission

Electron Microscopy, Molecular Mechanics, and Theory of Optical Spectra. *J. Phys. Chem. C* **2016**, *120*, 19416–19433.

(6) Berendsen, H. J. C.; van der Spoel, D.; van Drunen, R. GROMACS: A Message-Passing Parallel Molecular Dynamics Implementation. *Comput. Phys. Commun.* **1995**, *91*, 43–56.

(7) Abraham, M. J.; Murtola, T.; Schulz, R.; Páll, S.; Smith, J. C.; Hess, B.; Lindahl, E. GROMACS: High Performance Molecular Simulations Through Multi-Level Parallelism From Laptops to Supercomputers. *SoftwareX* **2015**, *1-2*, 19 – 25.

(8) Wang, J.; Wolf, R. M.; Caldwell, J. W.; Kollman, P. A.; Case, D. A. Development and Testing of a General Amber Force Field. *J. Comput. Chem.* **2004**, *25*, 1157–1174.

(9) Patmanidis, I.; Wang, W.; de Vries, A. H.; Wassenaar, T. A.; Portale, G.; Marrink, S. J. Insights on the structure of C8S3 nanotubes with atomistic simulations and SAXS. *Manuscript in Preparation* **2020**,

(10) MacKerell Jr, A. D.; Nilsson, L. In *Computational biochemistry and biophysics*; Becker, O. M., MacKerell Jr, A. D., Roux, B., Watanabe, M., Eds.; CRC Press, 2001; pp 441–464.

(11) Wu, E. L.; Cheng, X.; Jo, S.; Rui, H.; Song, K. C.; DÃąvila-Contreras, E. M.; Qi, Y.; Lee, J.; Monje-Galvan, V.; Venable, R. M.; Klauda, J. B.; Im, W. CHARMM-GUI Membrane Builder toward realistic biological membrane simulations. *Journal of Computational Chemistry* **2014**, *35*, 1997–2004.

(12) Jorgensen, W. L.; Chandrasekhar, J.; Madura, J. D.; Impey, R. W.; Klein, M. L. Comparison of Simple Potential Functions for Simulating Liquid Water. *J. Chem. Phys.* **1983**, *79*, 926–935.

(13) Bussi, G.; Donadio, D.; Parrinello, M. Canonical sampling through velocity rescaling. *J. Chem. Phys.* **2007**, *126*, 014101.

(14) Berendsen, H. J. C.; Postma, J. P. M. v.; van Gunsteren, W. F.; DiNola, A.; Haak, J. R. Molecular Dynamics With Coupling to an External Bath. *J. Chem. Phys.* **1984**, *81*, 3684–3690.

(15) Tironi, I. G.; Sperb, R.; Smith, P. E.; van Gunsteren, W. F. A generalized reaction field method for molecular dynamics simulations. *J. Chem. Phys.* **1995**, *102*, 5451–5459.

(16) D'Avino, G.; Muccioli, L.; Zannoni, C.; Beljonne, D.; Soos, Z. G. Electronic Polarization in Organic Crystals: a Comparative Study of Induced Dipoles and Intramolecular Charge Redistribution Schemes. *J. Chem. Theory Comput.* **2014**, *10*, 4959–4971.

(17) Swart, M.; van Duijnen, P. T. DRF90: a Polarizable Force Field. *Mol. Simul.* **2006**, *32*, 471–484.

(18) Thole, B. T. Molecular polarizabilities calculated with a modified dipole interaction. *Chem. Phys.* **1981**, *59*, 341–350.

(19) van Duijnen, P. T.; Swart, M. Molecular and atomic polarizabilities: Thole's model revisited. *J. Phys. Chem. A* **1998**, *102*, 2399–2407.

(20) Michaud-Agrawal, N.; Denning, E. J.; Woolf, T. B.; Beckstein, O. MDAnalysis: a toolkit for the analysis of molecular dynamics simulations. *J. Comput. Chem.* **2011**, *32*, 2319–2327.

(21) Gowers, R. J.; Linke, M.; Barnoud, J.; Reddy, T. J. E.; Melo, M. N.; Seyler, S. L.; Dotson, D. L.; Domanski, J.; Buchoux, S.; Kenney, I. M.; Beckstein, O. MDAnalysis: a Python package for the rapid analysis of molecular dynamics simulations. Proceedings of the 15th Python in Science Conference, Austin, TX. Scipy, 2016; pp 102–109.

(22) Breneman, C. M.; Wiberg, K. B. Determining atom-centered monopoles from molecular electrostatic potentials. The need for high sampling density in formamide conformational analysis. *J. Comput. Chem.* **1990**, *11*, 361–373.

(23) Frisch, M. J.; Trucks, G. W.; Schlegel, H. B.; Scuseria, G. E.; Robb, M. A.; Cheeseman, J. R.; Scalmani, G.; Barone, V.; Petersson, G. A.; Nakatsuji, H.; Li, X.; Caricato, M.; Marenich, A. V.; Bloino, J.; Janesko, B. G.; Gomperts, R.; Mennucci, B.; Hratchian, H. P.; Ortiz, J. V.; Izmaylov, A. F.; Sonnenberg, J. L.; Williams-Young, D.; Ding, F.; Lipparini, F.; Egidi, F.; Goings, J.; Peng, B.; Petrone, A.; Henderson, T.; Ranasinghe, D.; Zakrzewski, V. G.; Gao, J.; Rega, N.; Zheng, G.; Liang, W.; Hada, M.; Ehara, M.; Toyota, K.; Fukuda, R.; Hasegawa, J.; Ishida, M.; Nakajima, T.; Honda, Y.; Kitao, O.; Nakai, H.; Vreven, T.; Throssell, K.; Montgomery, J. A., Jr.; Peralta, J. E.; Ogliaro, F.; Bearpark, M. J.; Heyd, J. J.; Brothers, E. N.; Kudin, K. N.; Staroverov, V. N.; Keith, T. A.; Kobayashi, R.; Normand, J.; Raghavachari, K.; Rendell, A. P.; Burant, J. C.; Iyengar, S. S.; Tomasi, J.; Cossi, M.; Millam, J. M.; Klene, M.; Adamo, C.; Cammi, R.; Ochterski, J. W.; Martin, R. L.; Morokuma, K.; Farkas, O.; Foresman, J. B.; Fox, D. J. Gaussian 16, Revision A.03. 2016; `http://gaussian.com/citation_a03/`, Gaussian Inc. Wallingford CT.

(24) Chai, J.-D.; Head-Gordon, M. Long-range corrected hybrid density functionals with damped atom–atom dispersion corrections. *Phys. Chem. Chem. Phys.* **2008**, *10*, 6615–6620.

(25) Hehre, W. J.; Radom, L.; v. R. Schleyer, P.; Pople, J. A. *Ab Initio Molecular Orbital Theory*; Wiley, New York, 1986.

(26) Runge, E.; Gross, E. K. U. Density-functional theory for time-dependent systems. *Phys. Rev. Lett.* **1984**, *52*, 997–1000.

(27) Bondarenko, A. S.; Patmanidis, I.; Alessandri, R.; Souza, P. C. T.; de Vries, A. H.;

Marrink, S. J.; Jansen, T. L. C.; Knoester, J. C8S3 molecular geometry and charge distributions. **2020**, doi: 10.6084/m9.figshare.12369335.v1.

(28) Czikkely, V.; Försterling, H. D.; Kuhn, H. Light Absorption and Structure of Aggregates of Dye Molecules. *Chem. Phys. Lett.* **1970**, *6*, 11–14.

(29) Didraga, C.; Pugzlys, A.; Hania, P. R.; von Berlepsch, H.; Duppen, K.; Knoester, J. Structure, Spectroscopy, and Microscopic Model of Tubular Carbocyanine Dye Aggregates. *J. Phys. Chem. B* **2004**, *108*, 14976–14985.

(30) Didraga, C.; Klugkist, J. A.; Knoester, J. Optical Properties of Helical Cylindrical Molecular Aggregates: The Homogeneous Limit. *J. Phys. Chem. B* **2002**, *106*, 11474–11486.
